# Response to Immunization against SARS-CoV-2 and Risk of Omicron Infection in Dialysis Patients: A Prospective Cohort Study

**DOI:** 10.3390/jcm12154983

**Published:** 2023-07-28

**Authors:** Johannes Werzowa, Martina Behanova, Ammon Handisurya, Florian Heger, Alexander Indra, Barbara Holzer, Thomas Dechat, Silvia Spitzer, Sandra Lederer, Daniel A. Kraus, Jochen Zwerina, Ruth D. E. Fritsch-Stork

**Affiliations:** 1 Ludwig Boltzmann Institute of Osteology at Hanusch Hospital of OEGK and AUVA Trauma Centre Meidling, 1140 Vienna, Austria; johannes.werzowa@oegk.at (J.W.); thomas.dechat@osteologie.lbg.ac.at (T.D.); silvia.spitzer@osteologie.lbg.ac.at (S.S.); daniel.kraus@osteologie.lbg.ac.at (D.A.K.); jochen.zwerina@osteologie.lbg.ac.at (J.Z.); 2 1st Medical Department, Hanusch Hospital, 1140 Vienna, Austria; ammon.handisurya@oegk.at (A.H.); sandra.lederer98@gmx.at (S.L.); 3 Austrian Agency for Health and Food Safety (AGES), 1090 Vienna, Austria; florian.heger@ages.at (F.H.); alexander.indra@ages.at (A.I.); 4 Institute for Veterinary Disease Control, Austrian Agency for Health and Food Safety (AGES), 2340 Moedling, Austria; barbara.holzer@fh-krems.ac.at; 5 Medical Center Mariahilf of OEGK, 1060 Vienna, Austria; ruth.fritsch-stork@oegk.at; 6 Medical Faculty, Sigmund Freud Private University, 1020 Vienna, Austria

**Keywords:** SARS-CoV-2, dialysis patients, Omicron, humoral response, cellular response

## Abstract

It is not well established to what extent previous immunizations offer protection against infections with the SARS-CoV-2 Omicron variant in dialysis patients. We aimed to define the relevant humoral response in dialysis patients using a SARS-CoV-2 IgG chemiluminescence microparticle immunoassay (CMIA) compared to the activity of neutralizing antibodies assessed by a virus neutralization test. Next, we aimed to determine differences in humoral and cellular response levels over time among patients infected or not infected by the Omicron variant of SARS-CoV-2. Immunological parameters of cellular and humoral response to SARS-CoV-2 were analyzed at baseline and after 3 (T3), 6 (T6) and 14 months (T14). In this monocentric cohort study, we followed 110 dialysis patients (mean age 68.4 ± 13.7 years, 60.9% male) for a median of 545 days. We determined an anti-SARS-CoV-2 IgG level of 56.7 BAU/mL as an ideal cut-off value with a J-index of 90.7. Patients infected during the Omicron era had significantly lower (*p* < 0.001) mean antibody levels at T0 (3.5 vs. 111.2 BAU/mL), T3 (269.8 vs. 699.8 BAU/mL) and T6 (260.2 vs. 513.9 BAU/mL) than patients without Omicron infection. Patients who developed higher antibody levels at the time of the basic immunizations were less likely to become infected with SARS-CoV-2 during the Omicron era. There is a need to adjust the cut-off values for anti-SARS-CoV-2 IgG levels in dialysis patients.

## 1. Introduction

Since the onset of the SARS-CoV-2 pandemic, new virus variants have emerged, such as Beta, Delta, and Omicron, with their respective subvariants. Under the selection pressure of increasing immunity in the population, SARS-CoV-2 is transforming towards higher infectivity and lower pathogenicity [1].

Patients with end-stage renal disease (ESRD) undergoing renal replacement therapy are at high risk for severe infections with SARS-CoV-2 [2] and vaccine response rates after mRNA-based vaccines are diminished compared to the general population [3]. During the first COVID-19 wave in the year 2020, the European Renal Association (ERA) reported a 28-day mortality of 25% in dialysis patients after SARS-CoV-2 infection [4]. Mortality of COVID-19 has significantly declined for the general population during the spread of the Omicron variant despite high numbers of infected individuals [5], but infection with SARS-CoV-2 still confers an increased mortality risk in dialysis patients [6].

The development of effective vaccines was a milestone in the struggle for disease control over SARS-CoV-2, offering a high level of protection against severe infections, especially in highly vulnerable populations. However, the establishment of the Omicron variant and its subvariants as globally dominant strains has raised doubts about the effectiveness of the currently available vaccines due to their ability to escape the vaccine derived immunity [7]. In this respect, the increased infectivity of the Omicron strains, in combination with the waning and/or ineffective immunity of large parts of the population at the time of cessation of most protective measures, has led to high infection rates in many parts of the world during the year 2022.

It is not well established to what extent previous immunizations with vaccines originally developed against the initial SARS-CoV-2 variant and immunity through past SARS-CoV-2 infections offer protection against infections with the Omicron variant in dialysis patients. In particular, the potentially differential response of antibody-mediated and cellular host defense mechanisms remains to be elucidated.

The first aim of this study was to define the relevant humoral response observed in dialysis patients using a SARS-CoV-2 IgG CMIA (Abbott) compared to the activity of neutralizing antibodies assessed by a virus neutralization test as gold standard. The second objective was to determine differences in humoral and cellular response levels over time among patients infected or not infected by Omicron variant of SARS-CoV-2.

## 2. Materials and Methods

### 2.1. Study Design

This was a prospective observational monocentric cohort study conducted at a secondary center (Hanusch Hospital) in Vienna, Austria. All patients undergoing chronic hemodialysis (HD) or peritoneal dialysis (PD) at the study site in February 2021 (phase of enrollment) were considered eligible for participation in the present trial. Inclusion criteria were age > 18 years and terminal kidney disease on maintenance dialysis with HD or PD. Vaccine refusal was not an exclusion criterion.

Subjects, who gave written informed consent, were included in the study and followed from March 2021 to August 2022. Immunological parameters of cellular and humoral response to SARS-CoV-2 were analyzed at baseline and after 3 (T3), 6 (T6) and 14 months (T14; see below). Clinical parameters, including monthly biochemical measurement of, among others, blood cell count, inflammatory parameter, electrolytes and parameters of kidney and liver function, were performed over the complete study period. To assess the prevailing co-morbidities of the study participants, the Charlson Comorbidity Score was calculated [8]. According to legal regulations, all dialysis patients were screened weekly for SARS-CoV-2 infection using RT-PCR. Episodes of SARS-CoV-2 infection were recorded, including the date of RT-PCR positivity, disease severity and clinical outcome. At study end, the dates of patients’ SARS-CoV-2 positivity were compared with the date of occurrence of certain SARS-CoV-2 variants in Austria [9]. The most common variant at the respective timepoint was then assigned to the infected patients (Alpha, Delta, Omicron). The severity of SARS-CoV-2 infections were classified as mild (no need for hospitalization), severe (need for ventilation or intensive care) or fatal.

Patients who gave consent to being vaccinated against SARS-CoV-2 received the mRNA-based vaccine BNT162b2 (Pfizer-BioNTech, BioNTech Manufacturing GmbH, Mainz, Germany) or mRNA-1273 (Moderna-NIAID, MODERNA BIOTECH SPAIN, S.L., Madrid, Spain) according to the national vaccination recommendations. All patients and dialysis staff were obliged to wear FFP2 masks at all times on the dialysis ward and in the whole hospital area.

The study was conducted in accordance with the Declaration of Helsinki and approved by the local Ethics Committee (Ethikkommission der Stadt Wien EK 20-341-0121).

### 2.2. Study Size

Due to the exploratory nature of the study with no published data on SARS-CoV-2 vaccine response in dialysis patients at the time of study planning (December 2020), no hypothesis was intended to be tested. Rather, the study intended to generate new data on COVID-19 immunological response in dialysis patients. Therefore, no sample size calculation was performed.

### 2.3. Cell Lines and Viruses

Vero 76 clone E6 cells (CCLV-RIE929, Friedrich-Loeffler-Institute, Riems, Germany), used for the neutralization assays, were cultured in Eagle’s minimum essential medium (EMEM) with Hank’s balanced salt solution (HBSS, BioWhittaker, Lonza, Szabo Scandic, Vienna, Austria), supplemented with 10% fetal bovine serum (FBS, Corning, Szabo Scandic, Vienna, Austria). Vero E6 TMPRSS-2 cells (provided by Stefan Pöhlmann; Deutsches Primatenzentrum, Göttingen, Germany), initially described by Hoffmann et al. [10], were cultured in Dulbecco’s modified Eagle’s medium (DMEM) with 10% FBS and were used for virus propagation. The virus used for the neutralization assay was originally isolated from a clinical specimen (nasopharyngeal swab) taken in mid-March 2020 from a 25-year-old male patient in Lower Austria and further passaged twice on Vero E6 TMPRSS-2 cells (Clade: B.1, GISAID Accession ID EPI_ISL_583577).

### 2.4. Chemiluminescent Microparticle Immunoassay (CMIA)

Serum was analyzed for qualitative and quantitative determination of IgG antibodies against SARS-CoV-2 using the SARS-CoV-2 IgG II CMIA assay (Abbott GmbH, Vienna, Austria) according to the manufacturer’s instructions. As antibody levels were primarily determined in Abbotts AU/mL, values have been converted to more commonly used international antibody-binding units per milliliter (BAU/mL) by using a multiplication factor of 0.142 according to Abbott’s instructions.

### 2.5. Interferon-γ Release Assay (IGRA)

The QuantiFERON SARS-CoV-2 assay (Qiagen GmbH, Hilden, Germany), a SARSCoV-2 spike (S) protein-specific interferon-γ (IFN-γ) release assay (IGRA), was employed for assessing cell-mediated immune response by qualitative detection of IFN-γ produced by CD4^+^ and CD8^+^ T cells using enzyme-linked immunosorbent assay (ELISA). Assays were processed according to the manufacturer’s instructions.

### 2.6. Virus Neutralization Test

A neutralization assay was set up in flat-bottom 96-well tissue culture plates. Human sera were heat-treated for 30 min at 56 °C and diluted 1 in 4 in serum-free medium to a total volume of 50 µL. Sera were serially diluted twofold, with dilutions ranging from 1:4 to 1:512. Equal volumes of 50 μL SARS-CoV-2 with 100 TCID_50_, determined by the Reed and Muench method [11], were incubated at 37 °C for 90 min. Next, 25,000 Vero 76 clone E6 cells were added to each well, in a volume of 100 µL in EMEM supplemented with 10% FBS, and incubated for 4 days at 37 °C, 5% CO_2_ in a humidified incubator. All samples were set up in triplicates. The cytopathic effect (CPE) in every well was observed under an inverted optical microscope and the reciprocal of the highest serum dilution that protected more than 50% of cells from CPE was defined as the neutralizing titer.

Methods used for immunological testing are listed in Table 1.

### 2.7. Statistical Methods

Characteristics of patients and clinical parameters were measured using frequencies and percentages for categorical variables and means and standard deviation for continuous variables. *p*-values were two-sided, and the statistical significance level was set at 0.05.

The optimal cut off value for SARS-CoV-2 IgG II CMIA assay (Abbott) was estimated from T3 data by calculation of Youden Index (J-index) and the respective ROC curve.

We used repeated measures generalized linear mixed models to determine differences in antibody levels over time among patients infected and not infected by the Omicron variant. The fixed effects were time (four time points), Omicron infection (yes/no) and their interaction. We estimated a separate mean for each time point and patient group.

Differences in the prevalence of an Omicron infection by selected risk factors were assessed by chi-square test.

Statistical analyses were conducted using IBM^®^ SPSS^®^ Statistics for Windows, version 28 (IBM Corp., Armonk, NY, USA).

## 3. Results

**Figure 1:**
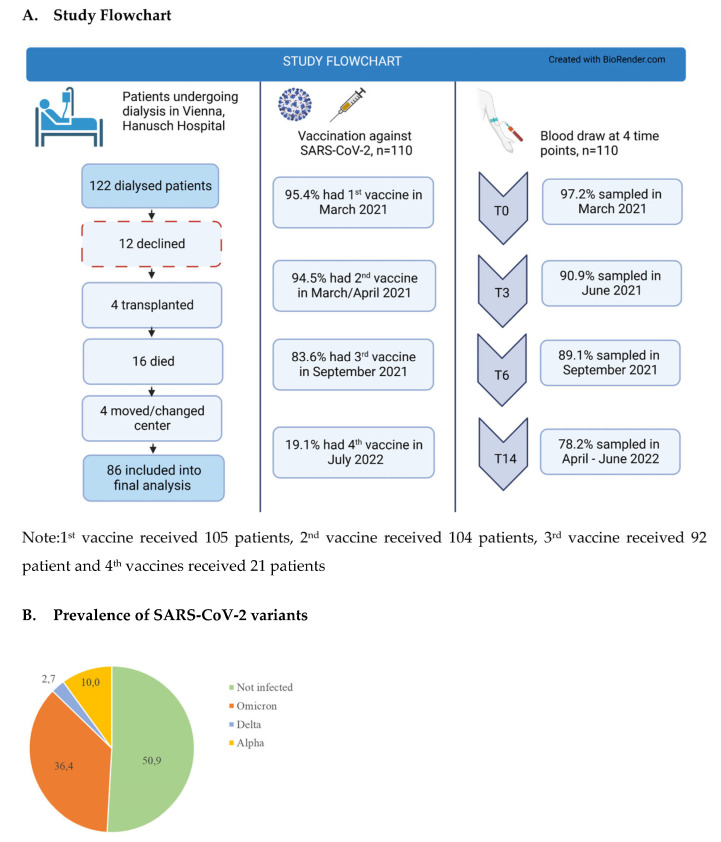
Overview of a study flowchart (**A**) and prevalence of SARS-CoV-2 variants (**B**).

Of 122 patients, in total, on maintenance dialysis at the study site, 110 subjects (105 HD, 5 PD) gave informed consent and were included in the present trial. The median follow-up time for these patients was 545 days (range 36–545 days). Eighty-six patients entered the final analysis at T14 (4 patients received a kidney transplant, 16 died, 4 were lost to follow-up due to a change of residence or of the dialysis center) (Figure 1A). During the study period, 49.1% of patients tested positive for SARS-CoV-2 (Figure 1B), the majority (36.4% of the total study population) after December 2021, the time when Omicron became the prevalent virus strain. Based on the prevalence data of the respective viral strains in Austria at that time, these patients were most likely infected with the Omicron variant of the virus (Figure 1B).

### 3.1. Patient Characteristics

Table 2 shows the patient characteristics at baseline and study outcomes. The mean age of patients was 68.4 years and 60.9% were male. Most patients (64.5%) received three vaccination doses during the study period and 19.1% received four or more doses; only 4.5% refused any vaccination. In most patients (72.2%), the course of the (first) SARS-CoV-2 infection was mild. Nevertheless, 20.4% experienced a severe course, requiring ventilatory support or intensive care. Three patients died (5.6%), two of them were unvaccinated. Five patients had SARS-CoV-2 infections twice, two of them were unvaccinated.

### 3.2. Antibody Titer over Time

**Figure 2:**
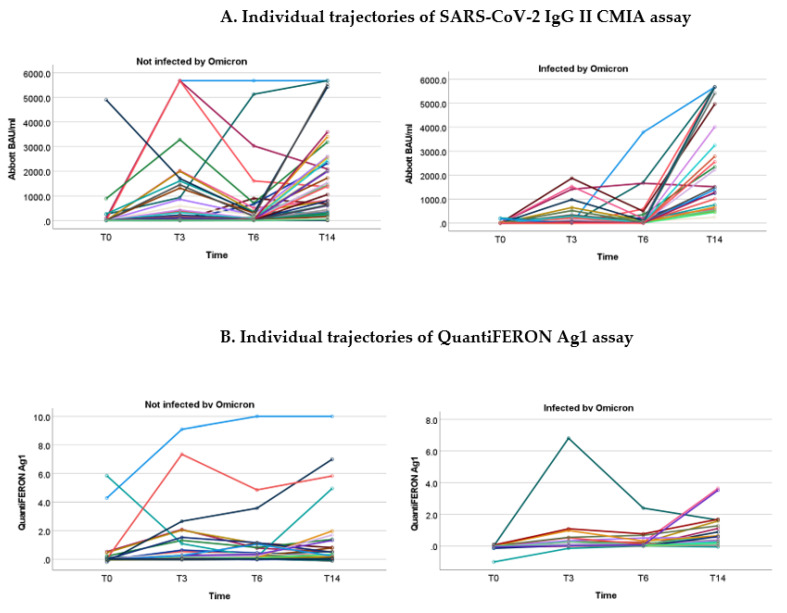
Plots of individual trajectories of SARS-CoV-2 IgG II CMIA assay (Abbott) (**A**) and QuantiFERON Ag1 (**B**) over time based on Omicron status. Each colored line represents one patient.

SARS-CoV-2 IgG antibody titers in dialysis patients increased over time, reaching peak values after 14 months (Figure 2A,B). Plots of individual trajectories of antibody titers over time show that patients with Omicron infection had lower levels of antibodies at T0, T3 and T6 compared to patients without infection. The effect of a booster vaccination led to high antibody levels, which were observed over a period of more than 6 months (Figure 2A, left panel).

**Figure 3:**
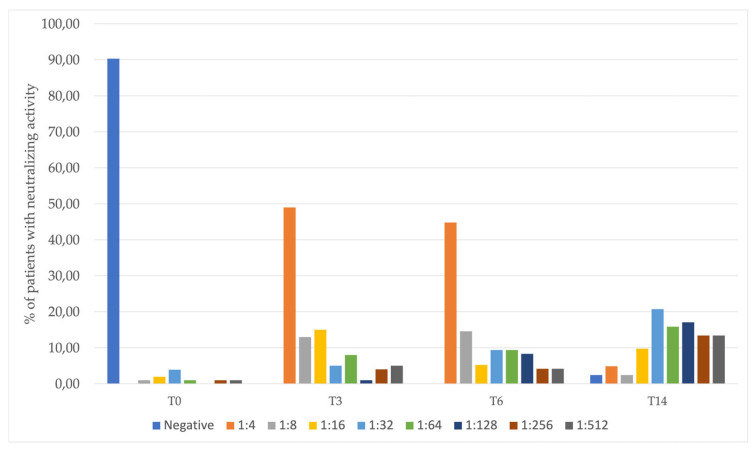
Proportion of patients with neutralizing antibody titers over time.

Results of the neutralization blocking assay are described in Figure 3.

### 3.3. Validity of Antibody Tests and Titers

**Figure 4:**
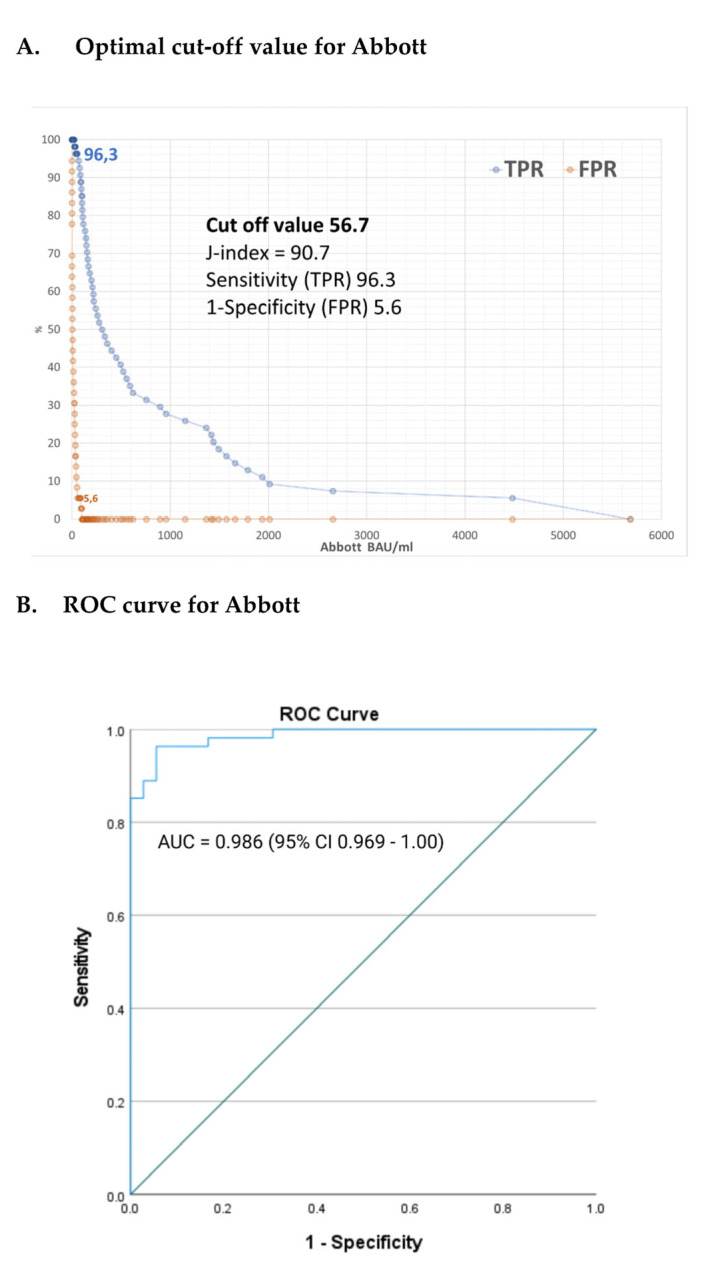
Cut-off values (**A**) and ROC curve (**B**) with the respective true positive rates (TPR, sensitivity) and false positive rates (FPR, 1-specificity) for Abbott three months after two doses of mRNA vaccines.

According to the manufacturer’s specifications, the cut-off for a reactive test result is defined as ≥7.1 BAU/mL. As the provided cut-off point was generated in non-ESRD patients, we compared the results of the antibody test to the neutralization test to verify the validity of the commonly available antibody test for SARS-CoV-2 in the vulnerable group of patients requiring maintenance dialysis. Figure 4 shows the rates of true positive and false positive results of the humoral immunity against SARS-CoV-2 as a function of anti-SARS-CoV-2 IgG levels (Abbott). True positive is defined as presence of virus-neutralizing activity and false positive as absence of virus-neutralizing activity in the patient’s serum. Interestingly, we determined a markedly higher anti-SARS-CoV-2 IgG level of 56.7 BAU/mL as the ideal cut-off value in our population of subjects on maintenance dialysis with a J-index of 90.7 (Figure 4A). At this level of SARS-CoV-2 IgG, the result had a 96.3% sensitivity and specificity of 94.4% (Figure 4B). By excluding patients with a positive neutralization test at T0, the results did not change with a cut-off of 56.7 BAU/mL.

Of note, we detected a positive neutralization result in 60% of patients (54 out of 90 patients with valid results) at T3 (time when majority of patients received two doses of vaccine). This increased to 95% (77 out of 81 patients with valid results) at T14 (when most patients had received three doses of vaccine and/or had been infected).

### 3.4. Differences in Antibody Levels over Time

Mixed models revealed statistically significant differences in mean antibody levels over time. Compared to baseline (T0), the mean differences of antibody level were −427.5 BAU/mL at T3 (95% CI −660.1, −194.9, *p* < 0.001), −329.8 BAU/mL at T6 (95% CI −566.1, −3.4, *p* = 0.007) and −2306.0 BAU/mL at T14 (−2721.3, −1890.8, *p* < 0.001) (Table 3).

Further, the interaction of time by Omicron event was statistically significant, with lower mean antibody levels at T0, T3 and T6 for patients infected by the Omicron variant (*p* < 0.001, respectively), suggesting a protective effect of both applied vaccines against the Omicron variant (Table 3). For example, at T6, the mean value of antibodies for patients not infected with Omicron was 513.9 BAU/mL, whereas for infected patients it was 260.2 BAU/mL (*p* < 0.001). At T14, when most of the patients had been vaccinated three times and had overcome the Omicron infection, the mean antibody value for infected patients was 3149.2 BAU/mL and 1577.5 BAU/mL for not infected patients (*p* < 0.001).

Similar patterns were observed for the cellular response as evaluated by QuantiFERON Ag1. Patients infected with Omicron had lower levels of QuantiFERON Ag1 at T0, T3 and T6. The mean difference, however, did not reach statistical significance when compared to non-infected patients (Table 4).

In a sensitivity analysis, including age adjustment, our results did not change (results not shown).

### 3.5. Difference in the Prevalence of an Omicron Infection by Selected Risk Factors

Omicron infections were more prevalent in males than females (*p* = 0.03). There were no significant differences in the prevalence of Omicron infections by categories of smoking, diabetes mellitus, glucocorticoid therapy, immunosuppressive therapy or kidney transplantation (for all *p* > 0.05, Table 4). Neither age (*p* = 0.71) nor the Charlson Comorbidity Index (*p* = 0.10) showed any significant differences between patients with and without Omicron (Table 5).

## 4. Discussion

This study was undertaken to characterize the humoral and cellular responses and clinical courses during the Omicron period until August 2022 after mRNA vaccination against SARS-CoV-2 in our cohort of 110 dialysis patients. We compared antibody titers using the SARS-CoV-2 IgG II CMIA assay (Abbott) to a serum-neutralization assay and redefined the cut-off level in our cohort of dialysis patients. In line with previous reports, we saw an increase of titers after the first two vaccines waning after 3 months. The booster vaccination then led to a sustained high antibody titer for at least 9 months thereafter, representing the longest follow up to our knowledge. Higher antibody levels and cellular responses after basic immunizations were associated with a smaller risk of infection during the Omicron era. Males were more often infected by Omicron than females, suggesting that male sex is a potentially negative prognostic factor for infection.

An altered immune system is one of the major components determining the vaccination response, together with sex, age, genetic background, medication, environmental characteristics and vaccine-specific factors [12]. The immune system in renal disease is hampered in several aspects, including both the innate and adaptive immune systems [13]. These alterations lead to lower rates of seroconversion, lower antibody titers and a less sustained response after immunization compared with healthy controls, especially after hepatitis B vaccination [14,15]. Several reports have also demonstrated impairments in the humoral and cellular responses in ESRD patients after vaccination against SARS-CoV-2 [16]. These reports often employed commercially available kits, measuring antibodies to the receptor binding region (RBD) as surrogates for the neutralization capacity of these antibodies. In some studies a higher cut-off was used, in part based on a comparison with surrogate neutralization tests [17], in others, an arbitrarily adjusted cut-off was chosen [18]. In a study investigating the titer of anti-SARS-CoV-2 antibodies 6–8 weeks after the third vaccination with a mRNA vaccine in 60 hemodialysis patients and 65 healthy controls using the Elecsys^®^ Anti-SARSCoV-2 S test, a serum-neutralization test was used as gold standard. The authors determined a cut-off point almost twice as high as the cut-off value provided by the manufacturer [18]. Our study generated results using the Abbott platform, raising the cut-off value by a factor of 8. In previous reports employing the SARS-CoV-2 IgG II Quant assay, the cut-off level for HD patients was set arbitrarily to twice the value given by the manufacturer, which, according to our findings, might lead to an overestimation of the vaccination effect [19], underlining the need to adjust cut-off levels in dialysis patients by comparison to the gold standard of neutralization tests.

A pattern of antibody titers after SARS-CoV-2 vaccination has emerged in several case-control studies: lower antibody titers/seroconversion rates measured shortly after the two basic vaccinations in ESRD patients compared to healthy controls, which slightly increase over the following weeks, but do not reach the levels seen in the control population [13,20,21,22,23]. This is followed by a steep decline of antibody levels in dialysis patients 6 months after the second vaccination dose [23,24]. After a third (booster) vaccination, response rates up to 96% were observed [25,26], followed by a decline in titers after approximately 3–4 months [23]. On the other hand, SARS-CoV-2 infection alone (without vaccination) does not elicit a lasting IgG response against the virus, not even in healthy individuals, where IgG reactivity was lost after 90 days in a third of cases [27], underscoring the necessity of repeat vaccinations to create a durable antibody response.

In our study, we observed a strong decline 6 months after the second vaccination, when most of the patients received the third vaccination dose. The booster led to high antibody levels, which were observed over a period of more than 6 months, which is the longest follow up after the third vaccination to our knowledge in dialysis patients to date. Accordingly, a fourth vaccination might be postponed in most dialysis patients to at least 6 months after the booster vaccination. However, new variants may necessitate adjustments in time schedules, as well as vaccine types, due to immune evasion.

Data relating to the T-cell response are less abundant and show a more stable reactivity over time, with an increase after the booster vaccination [19,23,28] and a decline 3 months after the third dose [19]. In our study, we observed a similar response 3 and 6 months after the first vaccination cycle, followed by a significant increase after the booster vaccination, measured after at least 6 months. As we did not assess the T-cell response shortly after the third vaccination, no conclusion can be drawn concerning a possibly declining course. However, we did observe a significantly more robust reactivity than after the first vaccination cycle.

Neutralization of the highly transmissible Omicron variant after the booster vaccination has been demonstrated in non-dialysis patients [29]. Comparing different types of vaccinations against SARS-CoV-2, the authors concluded that using an mRNA-based vaccines for the third dose elicits a more robust response in terms of hospitalization, severity and death due to Omicron as compared to only two vaccinations [30]. In dialysis patients, the third vaccination also offered protection against infection and severe outcomes in a large retrospective cohort study from Canada [31]. In our study period, the number of infections during the Omicron period (8 months) was three times higher than during the Delta period (10 months), underscoring the high transmissibility of Omicron in dialysis patients. In a study by Montez-Rath et al., 7% of all dialysis patients in a nationwide US cohort were infected during the first six weeks of the Omicron wave, which is in a similar range as the 36% of our patient group who were infected during the 8-month Omicron period in Austria [32].

With respect to baseline characteristics, the risk of infection during the Omicron wave in our study was only significantly influenced by male gender. Comorbidities and immunosuppressive therapy had no influence on infection risk in our cohort. Age, immunosuppressive therapy and frailty have been described as risk factors for infection and death in dialysis patients during the first COVID-19 wave in 2020 [4]. Regarding the clinical risk factors for breakthrough infection during the Omicron period, Chinnadurai et al. did not find a correlation with age, immunosuppressive therapy or comorbidity [33], in line with our findings.

Seropositivity after vaccination alone has been shown to offer no protection against breakthrough infection during the Omicron wave in dialysis patients [34]. Given that the level of protection against breakthrough infection correlated with the height of SARS-CoV-2 antibody levels in our study, our data underscore that the magnitude of the antibodies, rather than mere seropositivity, informs the level of protection. Likewise, higher levels of interferon release in the QuantiFERON assay reduced the Omicron infection risk in our study, although this difference did not reach statistical significance.

We are aware of the limitations of our study. Firstly, it was a single-center study with a relatively small sample size and no control group. Secondly, there was a time delay between SARS-CoV-2 positivity and blood sampling, thus making it difficult to clarify the correlation between antibody response and protection against SARS-CoV-2 infection. Additionally, positive cases were not sequenced for the specific variant. However, with a reported prevalence of almost 100% (according to the official Austrian agency for health and food safety) throughout the 8 months designated as the Omicron period in this study, we do consider the designation as Omicron variant legitimate despite the lack of sequencing.

The study strengths include weekly screening for SARS-CoV-2 infection with PCR testing and monitoring of SARS-CoV-2 antibodies and T-cell-mediated immunity over 14 months with periodical sampling at four time points and a long follow up after the third vaccination. Altogether, our findings underscore the importance of the establishment of a basic immunity against SARS-CoV-2 via three vaccinations in dialysis patients, thereby potentially also offering protection against new variants of SARS-CoV-2. Following three vaccinations, most dialysis patients develop a robust antibody response for at least 9 months post-vaccination. Despite the more benign course of Omicron compared to previous variants, booster vaccinations may offer protection against emergent variants, but the ideal timing of these booster vaccinations remains to be determined. Higher levels of SARS-CoV-2 antibodies also appear to be protective in dialysis patients.

## Figures and Tables

**Table 1:** Overview of methods for immunological testing.

	Readout	Unit
RT-PCR	Presence of viral particles in upper respiratory tract	Positive or negative
CMIA	Levels of anti-SARS-CoV IgG	BAU/mL
Neutralization test	Virus-neutralizing activity of antibodies	Titer
IGRA	Interferon-γ release in response to virus (“cellular response”)	IU/mL

**Table 2:** Baseline characteristics and outcomes of participants (*n* = 110).

		Missing Values, *n* (%)
Age, Mean (SD)	68.4 (13.7)	
Sex, *n* (%)		
Male	67 (60.9)	
Female	43 (39.1)	
BMI category, *n* (%)		9 (8.2)
Underweight	1 (0.9)	
Normal	37 (33.6)	
Overweight	37 (33.6)	
Obesity	26 (23.6)	
Smoking status, *n* (%)		7 (6.4)
Current smoker	21 (19.1)	
Ex-smoker	29 (26.4)	
Non-smoker	53 (48.2)	
Diabetes mellitus, *n* (%)		4 (3.6)
Yes	50 (45.5)	
No	56 (50.9)	
Charlson Comorbidity Index, Mean (SD)	6.9 (2.5)	
CKD origin, *n* (%)		
Tubulointerstitial	2 (1.8)	
Glomerular disease	22 (20.0)	
Hereditary nephropathy	7 (6.4)	
Hypertension	17 (15.5)	
Diabetes mellitus	15 (13.6)	
Other systematic disease	3 (2.7)	
Miscellaneous renal disease	4 (3.6)	
Unknown	40 (36.4)	
Dialysis frequency, *n* (%)		
Once a week	1 (0.9)	
Twice a week	18 (16.4)	
Three times a week	86 (78.2)	
Peritoneal dialysis	5 (4.5)	
History of kidney transplant, *n* (%)		
Yes	12 (10.9)	
No	98 (89.1)	
Current immunosuppressive therapy, *n* (%)		
Yes	10 (9.1)	
No	100 (90.9)	
Vaccination doses against SARS-CoV-2, *n* (%)		
0	5 (4.5)	
1	1 (0.9)	
2	12 (10.9)	
3	71 (64.5)	
4 or more	21 (19.1)	
First infection with SARS-CoV-2, *n* (%)	54 (49.1)	
Severity of first infection		1 (1.9)
Mild	39 (72.2)	
Severe	11 (20.4)	
Death	3 (5.6)	
Second infection with SARS-CoV-2, *n* (%)	5 (4.5)	
Severity of second infection		
Mild	5 (100)	
Severe	0 (0)	
Death	0 (0)	
Infected during Omicron wave, *n* (%)	40 (36.4)	
Positive neutralization test *n* (%)		
T0	10 (9.1)	7 (6.4)
T3	54 (49.1)	20 (18.2)
T6	56 (50.9)	25 (22.7)
T14	77 (70.0)	29 (26.4)

**Table 3:** Generalized linear mixed model with repeated measures analysis: estimates of fixed effects with Abbott as dependent variable.

Parameter		Estimate	SE	df	t	*p*-Value	95% Confidence Interval	
							Lower Bound	Upper Bound
Intercept		3149.2	321.5	84.8	9.8	<0.001	2509.9	3788.5
T0		−3145.7	315.8	82.8	−10.0	<0.001	−3773.8	−2517.7
T3		−2879.4	329.6	90.6	−8.7	<0.001	−3534.2	−2224.6
T6		−2889.0	297.5	83.1	−9.7	<0.001	−3480.6	−2297.4
T14		Ref.						
No Omicron event		−1571.7	424.8	86.1	−3.7	<0.001	−2416.1	−727.2
Omicron event		Ref.						
T0 * No Omicron event		1679.4	417.6	83.7	4.0	<0.001	848.9	2509.8
T3 * No Omicron event		2001.7	435.4	91.7	4.6	<0.001	1136.9	2866.5
T6 * No Omicron event		1825.4	394.5	83.8	4.6	<0.001	1040.8	2609.9
							95% Confidence Interval for Difference	
Time		Mean Difference (I-J)	SE	df		*p*-value	Lower Bound	Upper Bound
T0	T3	−427.5	117.1	91.2		0.000	−660.1	−194.9
	T6	−329.8	119.2	104.4		0.007	−566.1	−93.4
	T14	−2306.0	208.8	83.7		0.000	−2721.3	−1890.8
T3	T6	97.7	98.7	89.09		0.325	−98.3	293.8
	T14	−1878.5	217.7	91.7		<0.001	−2310.9	−1446.1
T6	T14	−1976.3	197.2	83.8		<0.001	−2368.5	−1584.0
Time by Omicron infection		Mean values of Abbott		SE		df	95% Confidence Interval	
							Lower Bound	Upper Bound
T0	not infected	111.2		59.2		104.9	−6.2	228.6
	infected	3.5		78.5		105.7	−152.1	159.1
T3	not infected	699.8		142.5		89.4	416.7	982.9
	infected	269.8		175.2		88.4	−78.4	618.0
T6	not infected	513.9		138.8		102.0	238.7	789.2
	infected	260.2		167.9		99.1	−72.9	593.3
T14	not infected	1577.5		277.6		87.6	1025.8	2129.3
	infected	3149.2		321.5		84.8	2509.9	3788.5

Explanatory note: Asterisk (*) denotes “interaction by”. The fixed intercept value of 3149 is the mean Abbot value for patients infected by Omicron at time T14. The intercept for T0 is 3149.201–3145.739, and this is significantly different than for T14. The coefficient of 1571.652 represents the average decrease in Abbott for each patient category (infected vs. not infected by Omicron) for T14. The interaction estimates represent the difference in the slope for not infected by Omicron for T0, T3 and T6. Further, the interaction means that we are 95% confident that patients not infected by Omicron at T0 had the Abbott level of 848.9 and 2509.8 points higher compared to Omicron-infected patients. The positive signs of estimates and their significance means that patients not infected at each time point—T0, T3, T6—had greater Abbott values than infected patients.

**Table 4:** Generalized linear mixed model with repeated measures analysis: estimates of fixed effects with QuantiFERON Ag1 as dependent variable.

Parameter		Estimate	SE	df	t	*p*-Value	95% Confidence Interval	
							Lower Bound	Upper Bound
Intercept		0.71	0.27	99.45	2.67	0.01	0.18	1.24
T0		−0.67	0.24	97.61	−2.81	0.01	−1.14	−0.20
T3		−0.37	0.20	88.71	−1.84	0.07	−0.77	0.03
T6		−0.52	0.17	82.66	−3.13	0.00	−0.85	−0.19
T14		Ref.						
No Omicron event		0.25	0.35	103.69	0.71	0.48	−0.44	0.93
Omicron event		Ref.						
T0 * No Omicron event		−0.09	0.31	99.74	−0.29	0.77	−0.71	0.53
T3 * No Omicron event		0.04	0.27	90.46	0.14	0.89	−0.49	0.56
T6 * No Omicron event		0.17	0.22	84.61	0.77	0.45	−0.27	0.61
							95% Confidence Interval for Difference	
Time		Mean Difference (I-J)	SE		df	*p*-value	Lower Bound	Upper Bound
T0	T3	−0.36	0.14		101.3	0.013	−0.64	−0.08
	T6	−0.28	0.12		103.6	0.029	−0.53	−0.03
	T14	−0.71	0.16		99.74	<0.001	−1.02	−0.41
T3	T6	0.08	0.07		91.76	0.219	−0.05	0.22
	T14	−0.35	0.13		90.46	0.009	−0.62	−0.09
T6	T14	−0.44	0.11		84.61	<0.001	−0.66	−0.22
Time by Omicron infection			Mean values of QuantiFERON Ag1		SE	df	95% Confidence Interval	
							Lower Bound	Upper Bound
T0	not infected		0.20		0.11	105.72	−0.02	0.42
	infected		0.04		0.14	107.52	−0.24	0.33
T3	not infected		0.63		0.18	103.01	0.27	0.98
	infected		0.34		0.22	102.6	−0.10	0.79
T6	not infected		0.61		0.16	105.26	0.30	0.92
	infected		0.19		0.19	100.78	−0.19	0.57
T14	not infected		0.96		0.22	109.71	0.52	1.40
	Infected		0.71		0.27	99.45	0.18	1.24

Asterisk (*) denotes “interaction by”.

**Table 5:** Differences in the prevalence of an Omicron infection by selected risk factors.

Variable		Prevalence of Omicron Infection	*p*-Value
Sex	Male	45.5% (30/66)	0.03
	Female	24.4% (10/41)	
Smoking	Never smoker	37.7% (20/53)	0.92
	Current or ex-smoker	36.7 (18/49)	
Diabetes mellitus	No	40.0% (22/55)	0.48
	Yes	33.3% (16/48)	
Glucocorticoid	No	36.4% (36/99)	0.44
	Yes	50.0% (4/8)	
Immunosuppressive therapy	No	37.8% (37/98)	0.79
	Yes	33.3% (3/9)	
Kidney transplant	No	40.0% (38/95)	0.11
	Yes	16.7% (2/12)	
Age ^a^			0.71
Charlson Comorbidity Index ^b^			0.10

^a^ Difference in median age between infected and non-infected patients assessed by Mann–Whitney *U* test. ^b^ Difference in mean Charlson Comorbidity Index between infected and non-infected patients assessed by an independent sample *t*-test.

## Data Availability

Data are available on a Figshare at doi:10.6084/m9.figshare.23600037 accessed on 10 July 2023.
